# Incorporating global dynamics to improve the accuracy of disease models: Example of a COVID-19 SIR model

**DOI:** 10.1371/journal.pone.0265815

**Published:** 2022-04-08

**Authors:** Hadeel AlQadi, Majid Bani-Yaghoub

**Affiliations:** 1 Department of Mathematics and Statistics, University of Missouri-Kansas City, Kansas City, Missouri, United States of America; 2 Department of Mathematics, Jazan University, Jazan, Saudi Arabia; Centers for Disease Control and Prevention, UNITED STATES

## Abstract

Mathematical models of infectious diseases exhibit robust dynamics, such as stable endemic, disease-free equilibriums or convergence of the solutions to periodic epidemic waves. The present work shows that the accuracy of such dynamics can be significantly improved by including global effects of host movements in disease models. To demonstrate improved accuracy, we extended a standard Susceptible-Infected-Recovered (SIR) model by incorporating the global dynamics of the COVID-19 pandemic. The extended SIR model assumes three possibilities for susceptible individuals traveling outside of their community:

• They can return to the community without any exposure to the infection.

• They can be exposed and develop symptoms after returning to the community.

• They can be tested positively during the trip and remain quarantined until fully recovered.

To examine the predictive accuracy of the extended SIR model, we studied the prevalence of the COVID-19 infection in six randomly selected cities and states in the United States: Kansas City, Saint Louis, San Francisco, Missouri, Illinois, and Arizona. The extended SIR model was parameterized using a two-step model-fitting algorithm. The extended SIR model significantly outperformed the standard SIR model and revealed oscillatory behaviors with an increasing trend of infected individuals. In conclusion, the analytics and predictive accuracy of disease models can be significantly improved by incorporating the global dynamics of the infection.

## 1- Introduction

COVID-19 is the recent infectious disease caused by the severe acute respiratory syndrome novel coronavirus (SARS-CoV-2). Because the transmissibility of this virus is relatively high and the outbreaks remained undetected for several days, COVID-19 turned into a global pandemic. Almost all countries of the world have been exposed to this virus. Since January 2020, more 256 million individuals have become infected with the COVID-19. The infection has resulted more than 5 millions death as of December, 2021 [1]. Just in the US, the COVID-19 cases are over 48 millions and more than 786,000 deaths as of December, 2021 [2]. In order to reduce the spread of COVID-19, businesses, communities, and governments have implemented different control measures such as mandatory lockdowns, social distancing, avoiding crowded events, using face masks in public, and vaccination [3]. Nevertheless, control of COVID-19 remains a major issue in several parts of the world [4].

COVID-19 is mainly transmitted from human-to-human via direct contact with contaminated surfaces and through the inhalation of respiratory droplets from infected individuals [5]. About 97% of the infected individuals will recover after period ranging between one to four weeks. Therefore, the use of mathematical modeling seems to be an appropriate approach to study COVID-19 transmission dynamics.

Mathematical modeling of infectious diseases has increasingly become an essential tool for prevention, prediction, and control of infectious diseases [6–8]. Since 1760, when Daniel Bernoulli developed the first disease model of smallpox, numerous mathematical models have been utilized to study disease transmission dynamics, and to predict, assess, and control infectious diseases [9–12].The substance of mathematical modeling lies in formulating a set of mathematical equations that mimic reality [13]. Mathematical models have been evolved from small sets of ordinary differential equations to sophisticated compartmental models with several equations (see [14–16] for a review).

One of the simplest, yet powerful, disease models is the standard Susceptible-Infected-Recovered (SIR) model, which was first introduced by Kermack and McKendrick in a series of three papers [17–19]. In a standard SIR model, the host population is divided into susceptible, infected and recovered individuals, denoted by S(t), I(t) and R(t), respectively. These quantities track the numbers of individuals in each compartment over different time periods [20, 21]. The standard SIR model without birth and death is represented by the set of ordinary differential equations [22]:

$$\begin{array}{l} \frac{dS}{dt}\left(t\right)=-\beta S\left(t\right)I\left(t\right).\\ \frac{dI}{dt}\left(t\right)=\beta S\left(t\right)I\left(t\right)-\gamma I\left(t\right).\\ \frac{dR}{dt}\left(t\right)=\gamma I\left(t\right). \end{array}$$ (1)

Where *β* is the average number of susceptible individuals infected by one infectious individual per contact per unit of time (the transmission rate), and *γ* is the average number of infected individuals recovered per unit of time (recovery rate).

For decades, the standard SIR model has been extended to various forms by adding different compartments to suit the biological, spatio-temporal and social aspects of the disease dynamics or to study the impact of intervention strategies on the disease transmission dynamics in different communities [23, 24]. For instance, it has been extended to SIR models with diffusion [25], contaminated environment [26, 27], delay terms [28], several strains of infection [29], and multiple routes of infection [30].

Recently, several researchers utilized mathematical modeling to analyze, and predict the transmission dynamics of COVID-19 pandemic [31–33]. Dynamics of COVID-19 epidemic has been simulated using different versions of SIR or SEIR (susceptible, exposed, infected and recovered) models [31, 32]. The main modification include adding asymptomatic and symptomatic infection compartments [33], hospitalization compartment [34], and quarantined and isolated compartments [31]. These models are presumably able to predict and simulate the number of infected cases by taking into consideration the asymptomatic and symptomatic cases, deaths, needs of beds in hospitals, and effect of control measures and the interventions to decrease the number of cases.

The abovementioned extended SIR models contribute to the existing literature. However, they largely ignore the effects of global dynamics of infection on local communities. The presence of a global pandemic or a widespread infection can largely influence the dynamics of infection in a local community. Most communities are well-connected and the assumption that the disease exists only within the community is invalid [35].

There have been attempts to include the global dynamics in different SIR models [28, 36]. Nevertheless, such extended SIR models have several unknown parameters and poorly fit to data of host population. Due to lack flexibility and poor fitness to data, there is a need for develop SIR models that are more practical.

**Fig 1:**
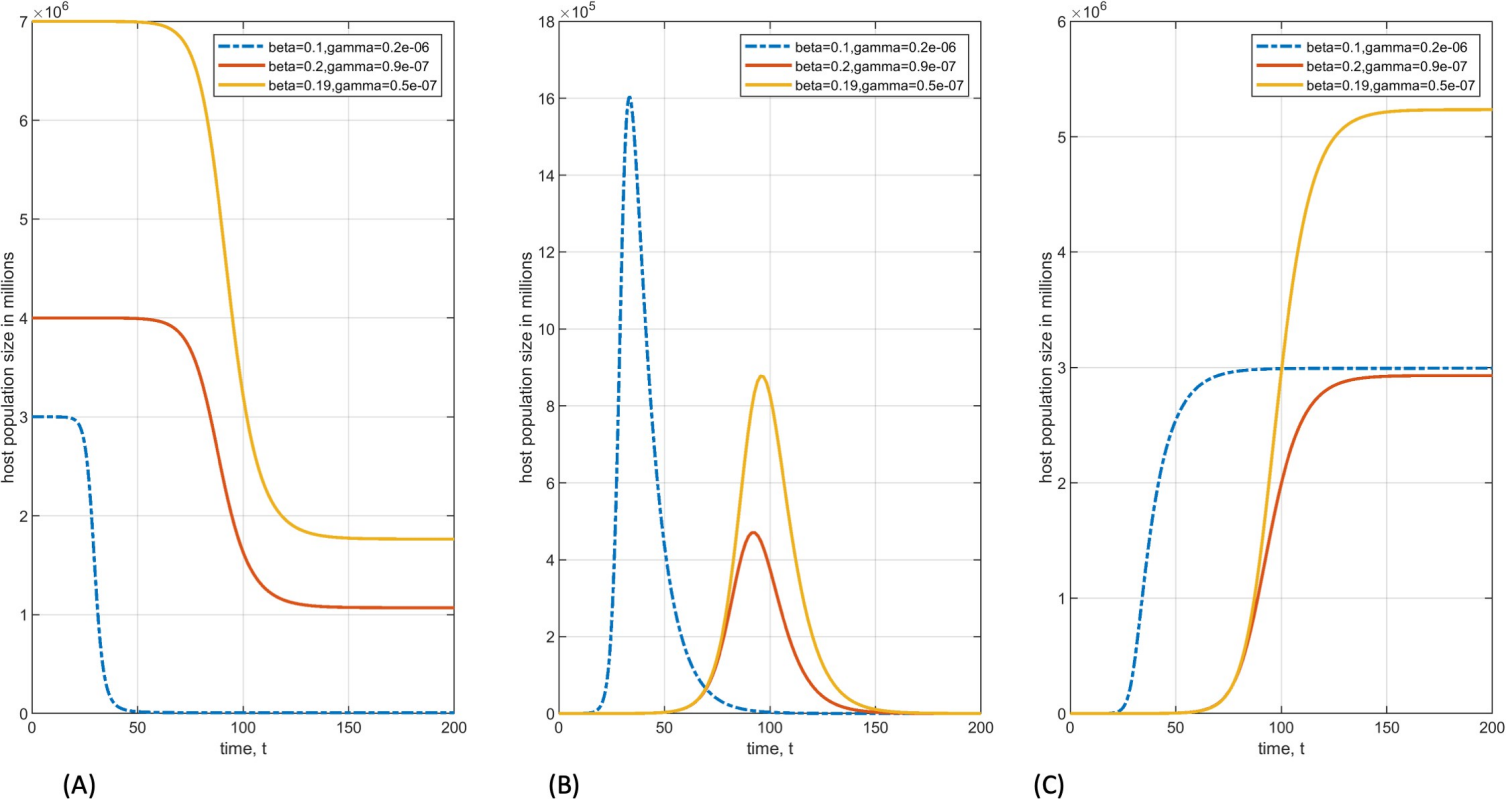
Qualitative behavior of the standard SIR model remains the same regardless of the parameter values (*β* and *γ*). (A) an inverted S-shape occurs for the susceptible population. (B) a bell-shaped epidemic wave of infected population. (C) a S-shaped curve of a recovered population.

Moreover, regardless of the parameter values, most numerical simulations of SIR models are limited to three distinct dynamics. The first of these dynamics is the solution curve of infected individuals may exhibit an epidemic wave before converging to a disease-free equilibrium [37], secondly, the solution curve of infected converge to an endemic equilibrium [38], the third of these dynamics is the solution curve of infected converge to periodic epidemic waves [39]. For the standard SIR model (1), the dynamics are even more limited. Namely, the solution curves always represent the same qualitative dynamics: an epidemic wave of the infectious population, an inverted S shape for susceptible population, and S shape for the recovered population. Regardless of the set of parameter values and initial conditions, such qualitative behaviors will always remain the same (see panels Fig 1A–1C). A quick review of the number of individuals infected with COVID-19, at the country [1], State [40], or city level [41], shows that the dynamics of COVID-19 is more complicated than a single epidemic wave. Therefore, it is essential to include the global dynamics of infection in a disease model.

The present work aims to address this issue. We extend the SIR model to a new model that includes the global impacts of the infection and is also capable of fitting well to infectious disease data. To incorporate the global effect and test the predictive accuracies of the extended model, we selected randomly six cities and states influenced by the COVID-19 global pandemic: Kansas City, Saint Louis, San Francisco, Missouri, Illinois, and Arizona.

We assumed three possibilities to consider in the SIR modeling of COVID-19: susceptible individuals from a local community can travel in and out of their community without any exposure to COVID-19, they can be exposed to COVID-19 while traveling and develop symptoms after they return to their community, or they can be diagnosed with COVID-19 during their traveling and return to their community after recovery. In the next section we include all these three possibilities in the extended SIR model.

The rest of the paper is organized as follows. In section. 2, we introduce the extended SIR model and its formulation. We explain the methodology that was used to fit the extended SIR model to COVID-19 data. In section. 3, we present the results of our analysis based on the six cities and states. In section. 4, we provide a discussion of the main results and additional factors to consider in the modeling process.

## 2- Materials and methods

### 2.1 Data

The COVID-19 data used in this study were obtained from the health department of Kansas City, Saint Louis, San Francisco, Missouri, Illinois, and Arizona [42–45]. The data were dated from March 10, 2020, to March 7, 2021(a total of 363 days). We did not include data after March 7, 2021, because our model does not include the effects of vaccination and it would be inappropriate to include the data thereafter. Specifically, the data variables consisted of date, total number of cases, new cases, total deaths, new deaths, and total number of individuals tested for COVID-19.

We used abovementioned data to extract the daily number of recovered, susceptible, and infected individuals (see S1 File for the algorithms used for generating the data).

Table 1 provides the basic descriptive statistics of Kansas City, Saint Louis, San Francisco, Missouri, Illinois, and Arizona of daily COVID-19 data of infected individuals.

**Table 1:** Descriptive Statistics of Kansas City (KC), Saint Louis (SL), San Francisco (SF), Missouri (MO), Illinois (IL), and Arizona (AZ) daily COVID-19 data from March 10, 2020 to March 7, 2021.

	KC	SL	SF	MO	IL	AZ
Minimum	1	1	2	0	0	0
Maximum	4109	2201	4600	62456	168855	129240
Mean	1413.140	772.60	1305.50	18395.38	45387.31	31215.03
Median	1280	549	917	15793	27896	16242
Range	4108	2200	4598	62456	168855	129240
Standard Deviation	1137.49	565.87	1141.94	16777.20	42645.57	33702.66

Observe that the statistics of susceptible and recovered data are comparable (see S1 File for the basic descriptive statistics). However, the statistics of infected individuals are at a much lower scale. Details of time series and spatial clusters of COVID-19 infection in Kansas City have been provided in [46, 47].

To estimate the number of susceptible individuals, we assumed an average incubation period of 5 days for COVID-19 [48]. We also considered one day for obtaining the COVID-19 test results. Hence, all of those who were tested positive were susceptible from the beginning until 6 days prior to obtaining the test results. Also, we added the individuals who take the test, but their results were negative. These individuals had presumably high risk of getting infected and therefore susceptible. The number of infected individuals were calculated by considering an average infection period 14 days [49]. Hence, we cumulatively added of new cases for 14 days until they recovered.

### 2.2 Model formulation

**Fig 2:**
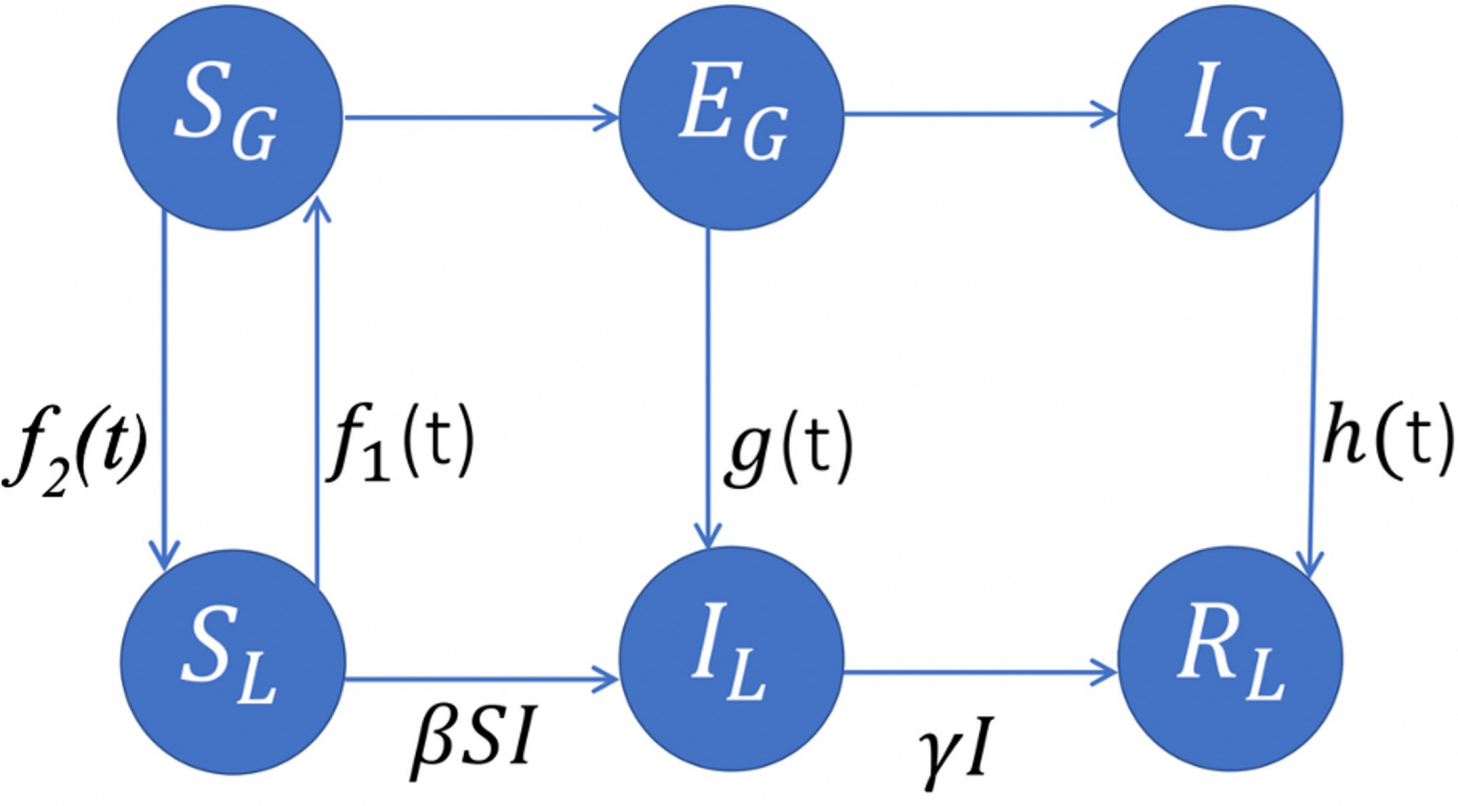
A schematic representation of the extended SIR model coupled with a global SEI model. L and G correspond to the number of individuals in the local and global communities, respectively.

We divided our population of N individuals living in a local community into sup-populations (i.e., compartments) of susceptible compartment S(t), infected compartment I(t), and recovered compartment R(t). As shown in Fig 2, the extended SIR model of COVID-19 transmission assumes three possibilities for susceptible individuals traveling outside of the community: They can return to the community without any exposure (the net rate is f(t) = f_2_(t)-f_1_(t)), they can be exposed COVID-19 and develop symptoms after returning to the community (the inflow rate of g(t)), or they can be tested positive during their trip and remain quarantined until fully recovered and thereafter return to the community (the inflow rate of h(t)). The extended SIR model is formulated by the following system of deterministic non-linear differential equations and Fig 2 gives the flow diagram of the model.

$$\begin{array}{l} \frac{dS}{dt}\left(t\right)=-\beta S\left(t\right)I\left(t\right)+f\left(t\right).\\ \frac{dI}{dt}\left(t\right)=\beta S\left(t\right)I\left(t\right)-\gamma I\left(t\right)+g\left(t\right).\\ \frac{dR}{dt}\left(t\right)=\gamma I\left(t\right)+h\left(t\right). \end{array}$$ (2)

where *β* and *γ* are the same parameters as in system (1). Functions *f*(*t*), *g*(*t*) and *h*(*t*) are differentiable and bounded functions and take into account the global effects of the infection. To avoid overfitting, our goal is to estimate *f*, *g* and *h* using least complicated forms.

Although adding exposed population can provide interesting dynamics, we decided to exclude the exposed compartment from our modeling. This is due to lack of data associated with exposed population. Namely, there is no known method of accurately identify the time series of exposed population in a community. In addition, the more compartments are added the harder it becomes to accurately estimate the parameter values. In some cases, the confidence intervals of estimated parameter values become extremely large due to high number of parameters and insufficient amount of data. With this rationale in mind, we therefore employ a two-step method to estimate the parameters of the model.

Individuals can get infected both within and outside of the community. A standard SIR model has been considered for progression of infection within the community. The extended SIR model coupled with a global SEI model where S_G_, E_G_, and I_G_ are the global of susceptible, exposed, and infected compartments respectively, and S_L_, I_L_, and R_L_ are the susceptible, infected, and recovered compartments in the local community, respectively. The extended model assumes three possibilities for susceptible individuals traveling outside of the community: They can return to the community without any exposure (the net rate is f(t) = f_2_(t)-f_1_(t)), they can be exposed to the infection and develop symptoms after returning to the community (rate of g(t)), or they can be tested positive during their trip and remain quarantined until fully recovered and thereafter return to the community (rate of h(t)).

### 2.3 Model fitting

The single-step numerical methods such as linearization and discretization [50] to estimate the parameter values of model (2) fail to converge, due to high degrees of freedom and unknown intervals of parameter estimations. We therefore proposed a two-step process for parameters estimation of model (2). First, we estimated the parameter values of the standard SIR model (1) and then we determined the functions f(t), g(t) and h(t) using the residual data of S(t), I(t) and R(t) subpopulations. As mentioned before, we used the COVID-19 data of susceptible, infected, and recovered individuals in Kansas City, Saint Louis, San Francisco, Missouri, Illinois, and Arizona for an epidemic period starting from March 10, 2020, to March 7, 2021.

In the first step, we numerically solved the system (2) using the MATLAB ode45 solver which is based on the fourth order Runge-Kutta method. The stability of the method is well established in [51]. For data fitting, the optimization function “fmincon” was used along with the common technique of the least-squares method [52, 53]. This method minimizes the sum of the squared residuals, that is, the difference between model predictions and their corresponding data values. The sum of the squared residuals is calculated using the formula below

$$\text{E}=\frac{1}{M}\sum_{I=1}^{M}{\left(y-y_{i}\right)}^{2}$$ (3)

where M represents the total number of data points considered for fitting and y and y_i_ represent the values predicted by the model and those from the data, respectively.

We estimated the SIR parameter values by considering the following factors. Several studies indicated that the COVID-19 transmission rate of infection was 0.5 [54, 55]. Hence, we set *β* = 0.5. Also, some studies assumed the average recovery period (i.e 1/ *γ*) is about 7 days [54, 55], which results in the initial value of *γ* = 0.13. Also, to be consistent with the data, we set our initial conditions to the number of susceptible, infected and recovered at = 1. The estimated model parameters are provided in Table 2.

**Table 2:** Estimated parameter values of model (2) based on data of Kansas City (KC), Saint Louis (SL), San Francisco (SF), Missouri (MO), Illinois (IL), and Arizona (AZ).

Parameter	Description	KC	SL	SF	MO	IL	AZ
*λ*1	Linear recruitment rate	100	99.9997	71.42	-20	600	-20
*λ*2	Constant recruitment rate	-0.2481	-0.3278	-0.0809	-1.32	8.62	-10
*β*	Transmission rate	2.9*10^−16^	5.49*10^−4^	4.9*10^−4^	8.23	8.22	3.998
*γ*	Recovery rate*	7.3*10^−15^	0.1275	1.97*10^−9^	1.003	1.002	0.59
p1	Linear flow of recovered	0.633	0.288	0.553	10.2	23.92	14.92
p2	Constant flow of recovered	-8.01	2.876	-15.17	-477.6	-1190	-729.9

* The estimated values for the recovery rates are different than those of the standard SIR model.

In the second step, we fitted the global effect of infection on the community by estimating functions f(t), g(t) and h(t) in model (2).

Although model selection can be done using Akaike’s Information Criteria (AIC) and Bayesian Information Criteria (BIC) methods, we used MATLAB curve fitting toolbox to measure the goodness of fit (adjusted R^2^, sum of the squared residuals, etc.) to find the optimal forms of functions f(t), g(t) and h(t). Specifically, the model fitting resulted in the following forms:

$$\begin{array}{l} f\left(t\right)=\lambda_{1}t+\lambda_{2}.\\ g\left(t\right)=a_{1}b_{1}\cos\left(b_{1}T+c_{1}\right)+a_{2}b_{2}\cos\left(b_{2}T+c_{2}\right)+a_{3}b_{3}\cos\left(b_{3}T+c_{3}\right).\\ h\left(t\right)=p_{1}t+p_{2}. \end{array}$$

## 3- Results

Using the COVID-19 data of each city and states, we estimated the functions corresponding to the global effects *f*(*t*), *g*(*t*) and *h*(*t*) in model (2) using the abovementioned two steps in Kansas City, Saint Louis, San Francisco, Missouri, Illinois, and Arizona. The estimated net rate for the susceptible individuals who can return to the community without any exposure is given by *f*(*t*) = *λ*_1_ *t* + *λ*_2_. The estimated net rate of the individuals who exposed to the infection and develop symptoms after returning to the community is given by *g*(*t*) = *a*_*1*_*b*_1_cos(*b*_*1*_T+*c*_*1*_)+ *a*_*2*_*b*_*2*_ cos(*b*_*2*_T+*c*_*2*_*)+ a*_3_*b*_3_cos(*b*_3_*T*+*c*_3_)., and the estimated net rate of the individuals who tested positive during their trip and remain quarantined until fully recovered and thereafter return to the community is given by h(t) = *p*_1_*t* + *p*_2._ All parameter estimations of step 1 and step 2 are summarized in Tables 2 and 3, respectively. From Table 2, we noticed that the transmission rate in the states; Missouri, Illinois, and Arizona are much higher than the cities; Kansas City, Saint Louis, and San Francisco, which is due to the larger population size of states and cities. Note that the estimated values for recovery rates are different than those of the standard SIR model because of the recovery rate is influenced by the global effects. In Table 3, there are three parameters, *a*, *b* and *c* where *a* represents the amplitude of each wave for each city or state, *b* represents the frequency of each wave, and *c* represents the phase shift of each wave. Also, the value of T represents the period of each wave. For instance, the state of Missouri and the city of Kansas City have almost the same of three periodic epidemic waves of COVID-19. One happens every two years, the other epidemic wave every four months and half, and the last wave every nine months. The periodic waves could be associated with several factors such as, traveling, major social events, infection prevention policies, changes to the coronavirus itself, and the increase of people who become susceptible because they have not developed some immunity. [56].

**Table 3:** Estimated parameter values of the global functions f(t), g(t) and h(t) based on data of Kansas City (KC), Saint Louis (SL), San Francisco (SF), Missouri (MO), Illinois (IL), and Arizona (AZ).

Parameters	KC	SL	SF	MO	IL	AZ
*a*_1_	2412	5763	1929	3.28*10^4^	8.69*10^4^	6.65*10^4^
*b*_1_	0.009	0.0149	0.005	0.009	0.0051	0.0142
*c*_1_	2.548	1.75	2.94	2.43	2.794	2.008
*T*1 = 2*π*/*b*1	698	421.5	1256	698	1231.4	442.2
*a*_2_	609.1	416.6	1.41*10^4^	7048	3.85*10^4^	3.007*10^5^
*b*_2_	0.046	0.05	0.037	0.048	0.031	0.027
*c*_2_	-1.81	3.58	-4.875	3.88	-3.53	-2.812
*T*2 = 2*π*/*b*2	136.5	125.6	169.7	130.8	202.6	232.6
*a*_3_	826.1	5072	1.42*10^4^	1.3*10^4^	2.03*10^4^	2.7*10^5^
*b*_3_	0.025	0.017	0.037	0.025	0.048	0.029
*c*_3_	3.64	-1.70	4.48	-2.4	-2.47	0.138
*T*3 = 2*π*/*b*3	251.2	369.4	169.7	251.2	130.8	216.5

Note: See the supplementary document for the goodness of fit (R^2^).

**Fig 3:**
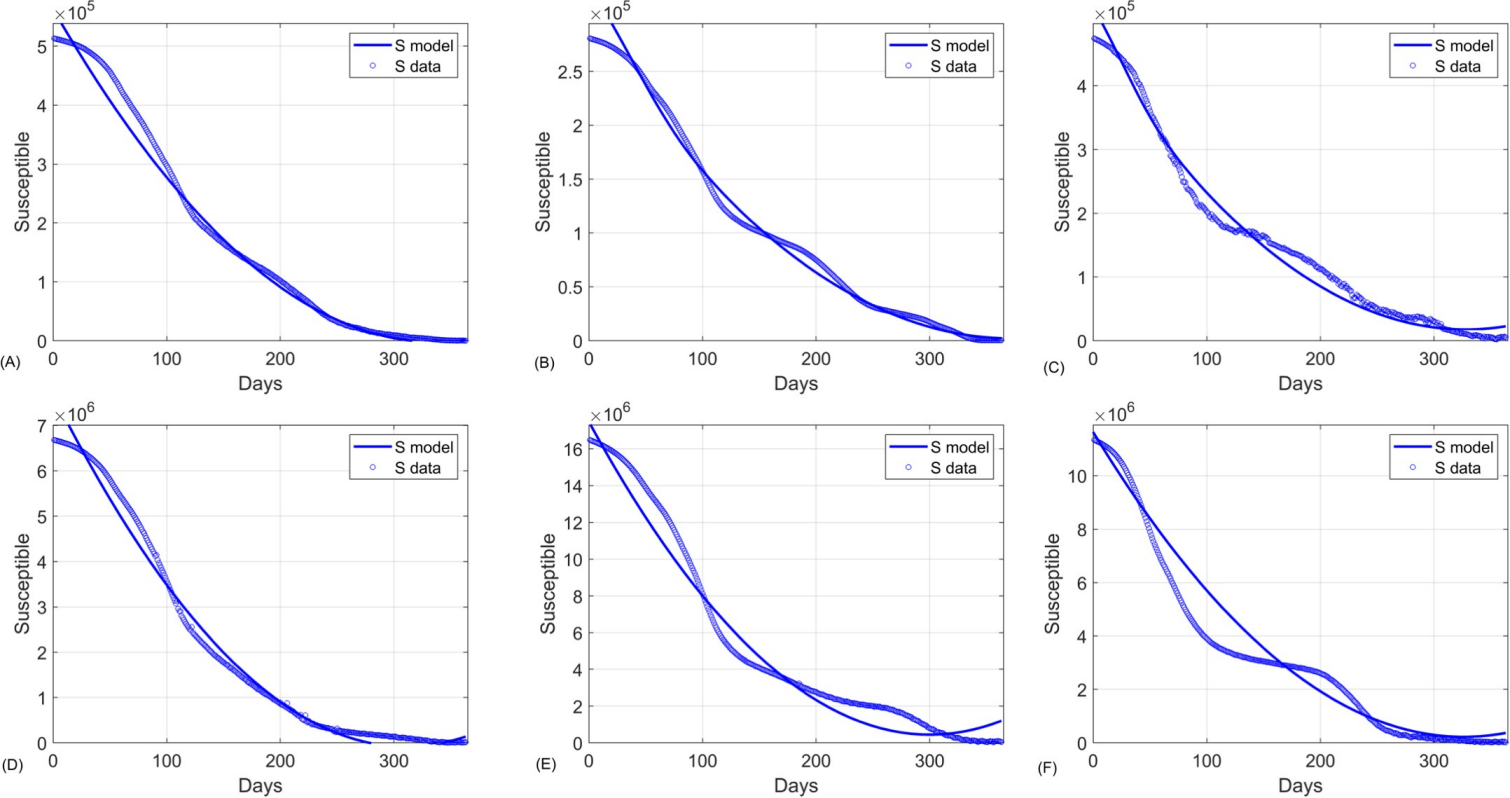
The extended SIR model fitted to the COVID-19 data of susceptible subpopulation in (A) Kansas City, (B) Saint Louis, (C) San Francisco, (D) Missouri, (E) Illinois, and (F) Arizona.

The panel of Fig 3A–3F shows that the extended SIR model of the susceptible solution curve fits well to the data of susceptible subpopulation in the cities and states. Likewise, S7 Table in S1 File shows the goodness of fit of susceptible subpopulation for Kansas City, Saint Louis, San Francisco, Missouri, Illinois, and Arizona data where R^2^ = 0.9909, 0.9911, 0.9802, 0.9878, 0.9761, 0.9583, respectively. (See S7 Table in S1 File).

**Fig 4:**
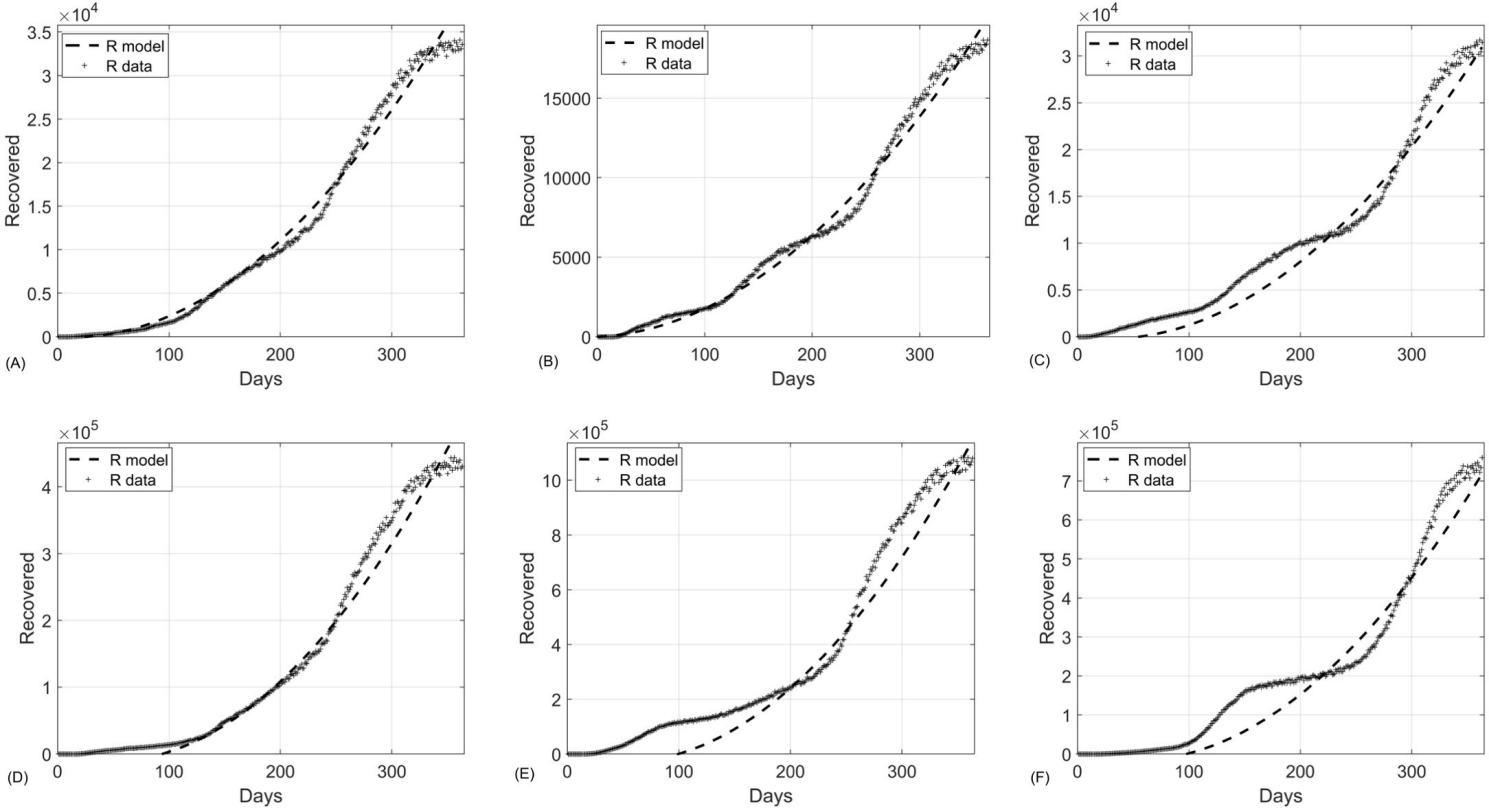
The extended SIR model fitted to the COVID-19 data of recovered subpopulation in (A) Kansas City, (B) Saint Louis, (C) San Francisco, (D) Missouri, (E) Illinois, and (F) Arizona.

Similarly, the panel of Fig 4A–4F shows the extended SIR model in the subpopulations of recovered individuals fits well to the data and have a goodness of fit R^2^ = 0.9873, 0.9893, 0.9804, 0.9844, 0.9748, 0.9606, respectively (See S7 Table in S1 File).

**Fig 5:**
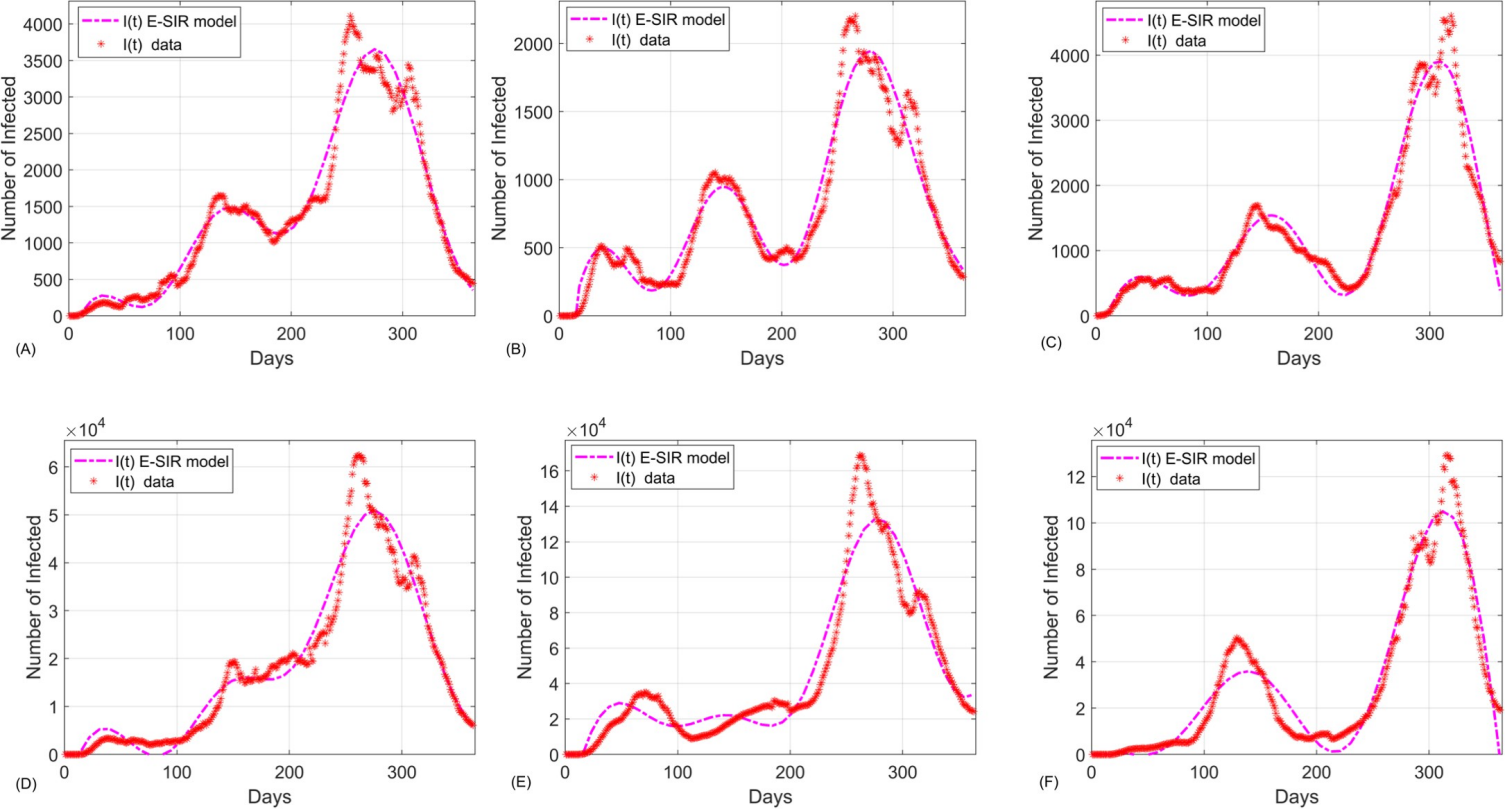
The extended SIR model fitted to the COVID-19 data of infected subpopulation in (A) Kansas City, (B) Saint Louis, (C) San Francisco, (D) Missouri, (E) Illinois, and (F) Arizona.

The panel of Fig 5A–5F shows that the dynamics of COVID-19 in the cities and states are more complicated than a single epidemic wave as common in the standard SIR model. The extended model is capable of revealing the underlying behaviors hidden in the data. Also, the extended SIR model in the subpopulations of infected individuals fits well to the data and have goodness of fit R^2^ = 0.9542, 0.9287, 0.962, 0.9438, 0.9015, 0.9492 for Kansas City, Saint Louis, San Francisco, Missouri, Illinois, and Arizona data, respectively. (See S7 Table in S1 File). Therefore, the inclusion of global effects to the SIR model can substantially improve the predictive accuracy of the model.

## 4- Discussion

**Fig 6:**
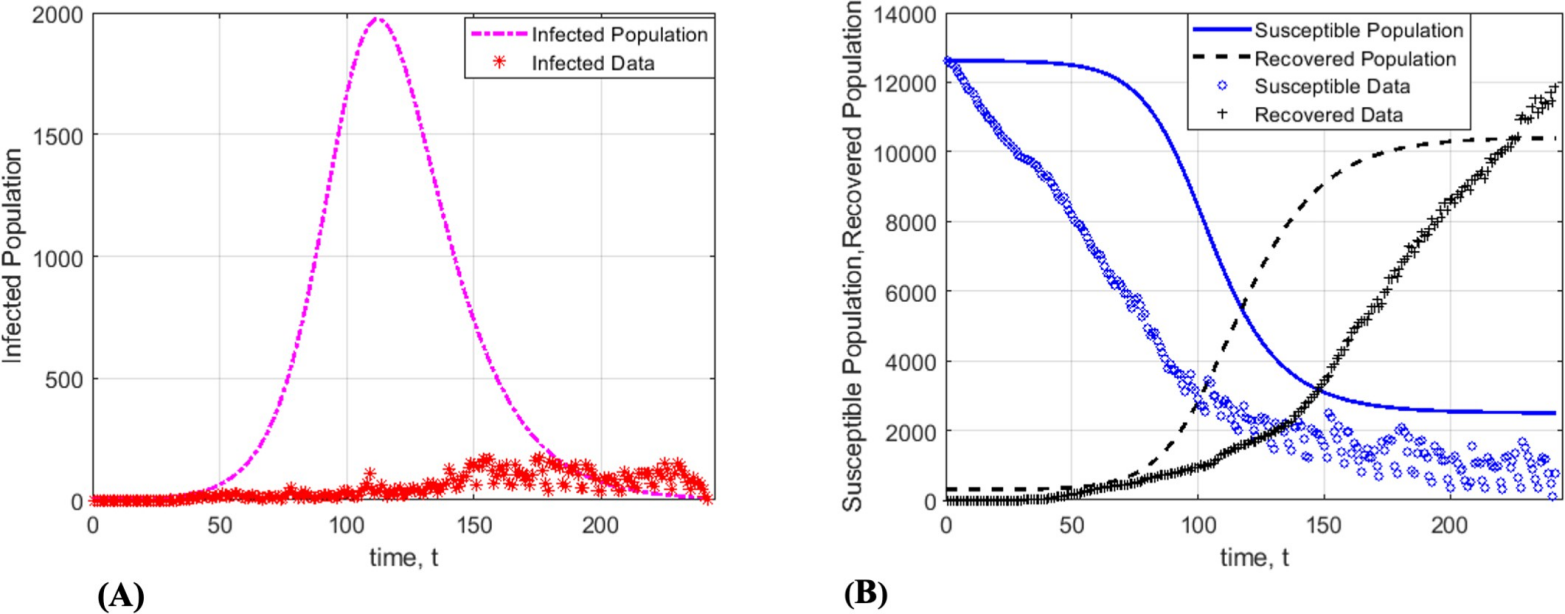
The standard SIR model fitted to fitted Kansas City COVID-19 data. (A) The standard SIR model poorly fits to the susceptible and recovered data. (B) The standard SIR model has poor fitness to data of infected population.

The present work highlights the importance of including global dynamics of infection in disease models to achieve higher predictive accuracies. We introduced a two-step algorithm for accurate estimation of infection parameters by considering both global and local effects of the infection spread in SIR models. The first step leads to estimation of local parameters (i.e., the transmission and recovery rates, *β* and, respectively) whereas the second step incorporates the global effects of the infection (i.e., estimation of functions *f*(*t*), *g*(*t*) and *h*(*t*)). To test the methodology, we applied the two-step model fitting algorithm to the extended SIR model (2) using Kansas City, Saint Louis, San Francisco, Missouri, Illinois, and Arizona data from March 10, 2020, to March 7, 2021. As shown in the panels of Figs 3–5, the two-step method resulted in solution curves that fit well to the COVID-19 data. The goodness of fit becomes more apparent when it is compared to that of the standard SIR model. Therefore, we compared the standard SIR model with the extended SIR model using the first 212 Kansas City COVID-19 data. As shown in Fig 6A and 6B, the solution curves of the standard SIR model poorly fit to the COVID-19. Moreover, Table 4 shows the comparisons of model fitness for the standard and extended SIR model. The extended SIR model of the susceptible solution curve has R^2^ = 0.9905 while in standard model R^2^ = 0.1551. Similarly the extended SIR model outperformed the standard SIR model in the subpopulations of recovered individuals (R^2^ = 0.9912 versus R^2^ = 0.47), and the subpopulation of infected individuals (R^2^ = 0.7083 versus R^2^ = -258.65). Note that the negative R^2^ value is because the classical SIR model does not follow the trend of the data.

**Table 4:** Comparisons of model goodness of fitness for the standard and extended SIR models.

Fitness	Extended	Standard
Corrected AIC	3.2799e+03	4.3130e+03
AIC	3.0351e+03	4.0689e+03
SSR (R^2^) for S	3.22e+09 (0.9905)	5.0377e+08 (0.1551)
SSR (R^2^) for I	4.1249e+05 (0.7083)	-1.5064e+08 (-258.65)*
SSR (R^2^) for R	3.51e+09 (0.9912)	1.6640e+09 (0.4700)

* The standard SIR model has an extremely poor fitting with respect to the infected individuals.

In addition to higher predictive accuracies of the extended SIR model (2), the solution curves revealed oscillatory behaviors with an increasing trend of infected individuals. This contrasts with the standard SIR model, where regardless of chosen parameter values, the solution curves always exhibit the same qualitative behaviors (see Fig 1A–1C).

Although the standard SIR has been proven useful to study local dynamics of various infections, it fails to capture the global effects of a widespread disease. The failure of standard SIR model to forecast the COVID-19 pandemic can be described by a variety of factors. One of these factors is that the standard SIR model assumes the population is closed, isolated, and ignores the effects of the global dynamics of infection on neighboring communities which is not a valid assumption [35]. Hence, by including the global infection effects in the disease models, we can identify underlying mechanisms governing the dynamics of infectious diseases.

In spite of several studies indicate that include temperature factor in the SIR model could potentially improve the model outcomes [57], recent studies suggested that the temperature has no influence on the propagation of the COVID-19 virus [58, 59]. In fact, some strains of the virus alter depending on their environments. They may live and grow in a variety of geographical areas or temperatures. Outside of laboratory tests, there is no way to anticipate how the virus would react in heat and humidity or even cold and dry temperatures.

The inability of the standard SIR model to fit there COVID-19 data has been identified by other researchers [60]. Nonetheless, the presence of a breakpoint due to strong policy interventions, mentioned in [60], does not necessarily reduce the prevalence of infection in a community dealing with a pandemic.

In conclusion, including the global dynamics of the infection and applying the two-step model fitting algorithm can enable us to extract vital information (e.g., presence of epidemic waves) from the data.

## Supporting information

S1 File Supplementary document. (DOCX)
