# Supplementary material for: Incorporating global dynamics to improve the accuracy of disease models: Example of a COVID-19 SIR model
Source: PLoS One. 2022 Apr 8;17(4):e0265815. doi: 10.1371/journal.pone.0265815 (PMC8993010; doi:10.1371/journal.pone.0265815)
Supplement: S1 File — (DOCX) [file pone.0265815.s001.docx]

**S1. The algorithm for generating infected and recovered data**

1. Data

The data we used for the extended SIR model were obtained from the Health Department of Kansas City, Saint Louis, San Francisco, Missouri, Illinois, and Arizona. The data were dated from March 10, 2020, to March 7, 2021(a total of 363 days). Specifically, the data consisted of six variable of date, total number of cases, new cases, total deaths, new deaths and total number of individuals tested for COVID-19. We used abovementioned data to extract the daily number of recovered and susceptible and infected individuals.

To estimate the number of susceptible individuals, we assumed an average incubation period is 5 days for COVID-19 [1]. We also considered one day for obtaining the COVID-19 test results. Hence, all of those who were tested positive were susceptible from the beginning until 6 days prior to obtaining the test results. Also, we added the individual who take the test, but their results were negative. These individuals had presumably high risk of getting infected and therefore susceptible. The number of infected individuals were calculated by considering an average infection period 14 days [2]. Hence, we cumulatively added of new cases for 14 days until they recovered. The algorithm to calculate the number of infected and recovered individuals as the following:

I(t) = $N_{i}$(1)+$N_{i}$ (2)+…+$N_{i}$ (14), where $N_{i}$ is the infected individual at time t, and t=14 days is the average time to get recovered from COVID-19 (3).

I(1)= $N_{i}$ (1)

I(2)= $N_{i}$ (1)+$N_{i}$ (2)

I(3)= $N_{i}$ (1)+$N_{i}$ (2)+$N_{i}$ (3)

…

I(14)= $N_{i}$ (1)+$N_{i}$ (2)+$N_{i}$ (3)+…+$N_{i}$ (14)

I(15)=$N_{i}$ (1)+ $N_{i}$ (2)+$N_{i}$ (3)+…+$N_{i}$ (14)+$N_{i}$ (15)-$N_{i}$ (1), where infected cases at time t=1 (i.e who get infected 14 days ago) is either recovered or died.

I(16)= $N_{i}$ (2)+…+$N_{i}$ (14)+$N_{i}$ (15)+$N_{i}$ (16)

So,

For i= 1:14

value = $N_{i}$ (i)

I(i) = Cumsum(value) (Cumulative infected cases from day 1 until day 14)

end

For i= 15:212

value = $N_{i}$ (i-13:i)

I(i) = Cumsum(value)

end

For i= 15:212

h= $N_{i}$ (1:i-15)

R(i)=random(95:99)*Cumsum(h)-Ndeath(i). Where is Ndeath(i) is cumulative death cases until day t.

In the algorithm, we assume the percentage of recovered case between (95%- 99%) of the cumulative infected individual of COVID-19 until day t. Then, we subtracted from the cumulative number of death individual until day t.

**S1 Table. Descriptive Statistics of Kansas City, Missouri daily COVID-19 data from March 10, 2020 to March 15, 2021**

|  | **Susceptible** | **Infected** | **Recovered** |
| --- | --- | --- | --- |
| Minimum | 1 | \| 1 \| \| --- \| | 3.63 |
| Maximum | 253055 | 418 | 33388.86 |
| Mean | 118601.41 | 101.720548 | 12992.06 |
| Median | 115547 | 79 | 9201.55 |
| Standard Deviation | 87126.9128 | 93.8125249 | 12012.59 |
| Range | 253054 | 417 | 33385.23 |

**S2 Table. Descriptive Statistics of St. Louis, Missouri daily COVID-19 data from March 3, 2020 to March 15, 2021**

|  | **Susceptible** | **Infected** | **Recovered** |
| --- | --- | --- | --- |
| Minimum | 1 | 0 | 0.90 |
| Maximum | 100499 | 251 | 18754.13 |
| Mean | 35294.4 | 54.19 | 7480.48 |
| Median | 28388 | 39 | 5948.61 |
| Standard Deviation | 30681.17 | 45.47 | 6273.94 |
| Range | 100498 | 251 | 18753.23 |

**S3 Table. Descriptive Statistics of San Francisco, California daily COVID-19 data from March 3, 2020 to March 15, 2021**

|  | **Susceptible** | **Infected** | **Recovered** |
| --- | --- | --- | --- |
| Minimum | 4 | 0 | 1.85 |
| Maximum | 1643808 | 561 | 31991 |
| Mean | 583213.60 | 91.78 | 11447.04 |
| Median | 443535.5 | 57 | 9373.5 |
| Standard Deviation | 525197 | 92.30 | 10111.63 |
| Range | 1643804 | 561 | 31989.15 |

**S4 Table. Descriptive Statistics of Missouri State daily COVID-19 data from March 7, 2020 to March 7, 2021**

|  | **Susceptible** | **Infected** | **Recovered** |
| --- | --- | --- | --- |
| Minimum | 23 | 0 | 0.92 |
| Maximum | 4565866 | 6346 | 447847.34 |
| Mean | 1766197.31 | 1316.83 | 149603.84 |
| Median | 1364523.5 | 1004 | 84019 |
| Standard Deviation | 1543442.31 | 1303.66 | 155729.18 |
| Range | 4565843 | 6346 | 447846.42 |

**S5 Table. Descriptive Statistics of Illinois State daily COVID-19 data from March 4, 2020 to March 7, 2021**

|  | **Susceptible** | **Infected** | **Recovered** |
| --- | --- | --- | --- |
| Minimum | 0 | 0 | 3.73 |
| Maximum | 18640190 | 15415 | 1110245.55 |
| Mean | 6211815.73 | 3247.51 | 380113.95 |
| Median | 4309941 | 1980 | 215210.61 |
| Standard Deviation | 5883615.78 | 3155.73 | 365143.84 |
| Range | 18640190 | 15415 | 1110241.82 |

**S6 Table. Descriptive Statistics of Arizona State daily COVID-19 data from March 4, 2020 to March 7, 2021**

|  | **Susceptible** | **Infected** | **Recovered** |
| --- | --- | --- | --- |
| Minimum | 33 | 0 | 1.81 |
| Maximum | 7908105 | 17234 | 759587.6 |
| Mean | 2490586.88 | 2239.71 | 234635.6 |
| Median | 1827793 | 1132 | 184042.15 |
| Standard Deviation | 2373491.59 | 2649.54 | 233107.15 |
| Range | 7908072 | 17234 | 759585.78 |

**S7 Table. Goodness of fit of Kansas City, Saint Louis, San Francisco, Missouri, Illinois, and Arizona data.**

| Fitness | **KC** | **SL** | **SF** | **MO** | **IL** | **AZ** |
| --- | --- | --- | --- | --- | --- | --- |
| R^2^ (Adjusted R^2^) for S | 0.9909  (0.9908) | 0.9911  ( 0.991) | 0.9802  (0.9801) | 0.9878  (0.9878) | 0.9761  (0.976) | 0.9583  (0.958) |
| R^2^ (Adjusted R^2^) for I | 0.9542  (0.9531) | 0.9287  (0.9271) | 0.962  (0.9611) | 0.9438  ( 0.9425) | 0.9015 (0.8993) | 0.9492  (0.948) |
| R^2^ (Adjusted R^2^) for R | 0.9873  (0.9872) | 0.9893  (0.9892) | 0.9804  (0.9803) | 0.9844  (0.9843) | 0.9748  (0.9747) | 0.9606  (0.9604) |

Refrences:

1. Qin J, You C, Lin Q, Hu T, Yu S, Zhou X-H. Estimation of incubation period distribution of COVID-19 using disease onset forward time: A novel cross-sectional and forward follow-up study. Sci Adv [Internet]. 2020 Aug 1;6(33):eabc1202. Available from: http://advances.sciencemag.org/content/6/33/eabc1202.abstract

2. Ramsay JO, Hooker G, Campbell D, Cao J. Parameter estimation for differential equations: a generalized smoothing approach. J R Stat Soc Ser B (Statistical Methodol [Internet]. 2007 Nov 1;69(5):741–96. Available from: https://doi.org/10.1111/j.1467-9868.2007.00610.x

.
